# Observed Shyness-Related Behavioral Responses to a Self-Presentation Speech Task: A Study Comparing Chinese and Canadian Children

**DOI:** 10.3390/bs14121147

**Published:** 2024-11-28

**Authors:** Xiaoxue Kong, Taigan L. MacGowan, Shumin Wang, Yan Li, Louis A. Schmidt

**Affiliations:** 1 Department of Psychology, University of Northern British Columbia, Prince George, BC V2N 4Z9, Canada; 2 Department of Psychology, Neuroscience & Behaviour, McMaster University, Hamilton, ON L8S 4L8, Canada; schmidtl@mcmaster.ca; 3 Department of Psychology, Queen’s University, Kingston, ON K7L 3N6, Canada; macgowat@mcmaster.ca; 4 Shanghai Institute of Early Childhood Education, Shanghai Normal University, Shanghai 201418, China; 1000529027@smail.shnu.edu.cn (S.W.); liyan@shnu.edu.cn (Y.L.)

**Keywords:** shyness-related behaviors, gender differences, cross-cultural comparison, cultural norms

## Abstract

Past research suggests that expressions of shyness are associated with several distinct behaviors that may differ between Eastern and Western cultures. However, this evidence has largely been derived from subjective ratings, such self-, teacher-, and parent-report measures. In this study, we examined between-country differences on measures of directly observed shyness-related behaviors during a speech task in children. Participants were 74 Chinese (*M*_age_ = 4.76 years old, *SD*_age_ = 0.62 years old; 77.0% male) and 189 Canadian (*M*_age_ = 4.80 years old, *SD*_age_ = 0.82 years old; 48.1% male) children aged 4–6 years. As predicted, the results reveal that Chinese children exhibit a higher frequency of gaze aversion and lower total time speaking compared to Canadian children. Additionally, significant interactions between country and gender were found for fidgeting and smiling behaviors, indicating that cultural expectations and norms influence how boys and girls express some shyness-related behaviors in social situations. These preliminary findings extend prior cross-cultural research on shyness-related behaviors indexed using subjective report measures to directly observed measures, highlighting the importance of cultural context in shaping children’s responses to social evaluation.

## 1. Introduction

Shyness is a characteristic of personality that reflects a tendency to feel anxious and inhibited in anticipation of or during social situations [1]. Shyness is perceived differently across Western and Eastern cultures due to varying cultural norms and values. In Western cultures, which emphasize individualism and personal freedom, shyness is often associated with social withdrawal and is linked to negative outcomes, such as low social status, peer victimization, and academic underachievement [2,3,4]. On the other hand, Eastern cultures, which prioritize group harmony and respect for authority, may view shyness more positively. In societies like traditional China, shyness is seen as a reflection of humility and respect for others, and is often considered an acceptable and adaptive behavior [5,6]. Although many previous articles have focused on the differences in shyness between Western and Eastern cultures [7], most studies have primarily measured shyness using subjective scales, with very few cross-cultural studies examining the expression of shyness-related behaviors.

Shyness-related behavioral responses occur when a person is in a situation that elicits perceived social evaluation, such as during a public performance or when meeting new people. Some of these behaviors include fidgeting, avoidance, gaze aversion, limited time spent speaking, and smiling [8,9,10,11]. The expression of shyness-related behaviors may vary by culture. For example, during a free-play laboratory session, Rubin and colleagues found that young children from East Asian countries (e.g., South Korea and China) exhibited more inhibitory behaviors, such as more time spent in contact with the mother and long latency to approach strangers and touch unfamiliar objects than young children from individualistic cultures (e.g., Italy and Australia) [12]. In addition, a study examined behavioral differences in trait measures of social anxiety, which is conceptually and empirically linked to shyness between Asian Americans and White Americans [13]. The responses of both groups of participants to social stimuli could be behaviorally coded in a speech task (e.g., avoidance of gaze and fidgeting), and the results indicate that Asian Americans reported more anxiety than White Americans on trait measures and mood rating scales.

Another important factor related to different manifestations of shyness in different cultures might be the social expectations of shyness vary across cultures [14]. Many Western studies consider shyness as a negative personality characteristic and consistently observe that young children lacked social initiation and exhibited more social withdrawal and inhibitory behaviors [15,16]. Since shyness may affect children’s interaction with their peers, a lack of social initiation may result in peer rejection and refusal [17], as well as deficits in long-term social competence [18]. In contrast, Chinese cultures value personal interdependence, focusing on group harmony associated with modesty, self-control, and unassuming behaviors [7,19]. These different social expectations influence the prevalence of shyness and how others treat shy people [7]. To gain social acceptance, connectedness, and a sense of belonging, children need to maintain or change their behavior in accordance with social views and expectations [12]. Additionally, China has experienced large-scale economic and social reforms in the past 30 years; different evaluations and perceptions of shyness are beginning to emerge [20]. In traditional Chinese culture, shyness has been viewed more positively than in Western cultures. However, in recent decades, as China has become more globalized and individualistic traits have gained prominence, perceptions of shyness have shifted [21,22]. Researchers found that shyness is increasingly seen as a social hindrance in urban China, and shyness may lead to more negative peer relations and psychological challenges compared to their rural counterparts [22]. Due to the sociocultural changes and shifts in attitudes toward shyness, the expression of shyness in both Eastern and Western contexts in the new era needs more attention and research.

Collectively, in addition to those behaviors associated with shyness and related constructs, such as social anxiety, which have been mentioned above, a question remains: Are there other behaviors (fidgeting, avoidance, gaze aversion, smiling, and length of speech) associated with shyness across countries? Whether these behaviors manifest with different intensities in different cultures is still in need of study. An investigation of these issues will help developmental researchers better understand the similarities and differences in the process of social interaction in different cultures. Additionally, most existing studies on cultural differences regarding the expression of shyness focus exclusively on toddlers and subjective measures of shyness (e.g., self-, parent, and teacher reports), leaving a gap in our understanding of whether these differences persist in preschoolers and early school-aged children in both Western and Eastern cultural contexts, and whether the effects are present on more objective behavioral measures of shyness. The preschool and early school-age periods are particularly critical for examining shyness, as these time periods are when key features of shyness, such as self-awareness and self-consciousness, begin to develop and crystallize [23].

While some cultural differences in shyness-related behaviors have been explored, it is also important to consider how these differences interact with gender, particularly given the varied social norms and expectations that shape behavior in different cultural contexts. Although there has been a general lack of consistent gender differences reported in observed shyness-related behaviors in early and middle childhood [15,24,25,26], cultural norms regarding gender roles may influence these behaviors differently across cultures. For example, Chinese culture has more traditional attitudes toward girls [27]. In traditional Chinese culture, compared to boys, girls are typically expected to be quieter, more modest, and gentler and softer [28]. These expectations can lead to greater demands on girls’ behavior, such as maintaining a proper posture, displaying good manners, and minimizing outward emotional expressions [29]. Research suggests that girls are often encouraged to be more compliant and to conform to social norms within family and social environments [30]. These cultural expectations may contribute to differences in how shyness is expressed and perceived across genders in different cultural contexts.

### The Present Study

The purpose of the current study was to examine whether expressions of shyness-related behaviors in response to a self-presentation birthday speech differed between preschoolers and early school-aged children in China and Canada. This task, in which children speak about their most recent birthday, is commonly used to elicit self-presentation anxiety and expressions of shyness-related behaviors in the lab in children [31,32,33]. Here, we coded five shyness-related behaviors in response to the task, including fidgeting, avoidance, gaze aversion, time spent speaking, and smiling. Based on previous findings regarding behavioral inhibition (a shyness-related construct) across different countries [18], we predicted that there would be between-country differences in observed shyness-related behaviors during the speech task. Specifically, we expected that Chinese children would exhibit more fidgeting, avoidance, gaze aversion, and would speak and smile less than Canadian children.

Although gender differences regarding shyness appear during early and middle childhood in the Western context [15,24], we know relatively little about the interaction between culture and gender in shyness-related behaviors. However, based on the previous literature and cultural differences, we hypothesized that the interaction between culture and gender may have a more noticeable impact on behaviors like fidgeting and smiling, as these are more influenced by gender-specific social norms [25,26]. In contrast, behaviors like avoidance, gaze aversion, and speaking duration reflect more general cultural expectations and may be less likely to show an interaction between culture and gender.

The practical implications of this study are relevant for understanding the socialization processes and cultural expectations that shape early childhood development across different societies. Insights from this research could inform culturally responsive approaches in educational and developmental settings, allowing caregivers and educators to support diverse expressions of shyness in constructive and culturally sensitive ways.

## 2. Materials and Methods

### 2.1. Participants

Chinese participants included 74 children (*M*_age_ = 4.76 years old, *SD*_age_ = 0.62 years old; 77.0% male) between four and six years of age and their parents who were recruited from preschools in Shanghai, the People’s Republic of China. The annual income of the Chinese sample, who were predominantly from the middle class in China, was approximately CAD 29,500. Procedures for the Chinese sample were conducted in a quiet extracurricular activity room in the kindergarten with a portable camera.

Canadian participants included 189 children (*M*_age_ = 4.80 years old, *SD*_age_ = 0.82 years old; 48.1% male) between four and six years of age and their parents who were recruited from a Child Database at McMaster University. The majority of the participants (81.9%) was White, and more than half (57.3%) had a total annual family income more than CAD 100,000 (in Canadian dollars). The Canadian sample was tested in a quiet playroom at McMaster University equipped with two closed-circuit TV (CCTV) cameras mounted on the walls.

Written informed consent was obtained from all children and their parents in both countries. In the consent form and prior to the study, both children and parents were informed about the entire study procedure and were told that they could withdraw from the experiment at any time for any reason during or after the study. If the parents chose to provide a debriefing to their children, the researchers would provide a debriefing of the study afterward. The Shanghai Normal University and McMaster University Research Ethics Boards approved the study procedures for both samples.

### 2.2. Self-Presentation Task

#### 2.2.1. Procedures

The procedures and measures were identical across the two countries. For both country samples, a self-presentation task was used to elicit shy behavior in preschool-aged children [31,32,33]. The children were asked to stand in front of a video camera, then to look into the camera and talk about their most recent birthday. They were informed that the videotape of their speech would be shown to other children later, so that other children could know all about what they did on their last birthday.

If children were silent for more than 20 s, experimenters prompted the child with open-ended questions about their last birthday (e.g., “Did you have a birthday cake?”). The speech was videotaped, and behavioral responses to the task were coded offline by research assistants who were naive to the study hypotheses.

#### 2.2.2. Behavioral Coding and Measures

Five shyness-related behaviors were coded based on coding schemes previously used with similar tasks [32,34,35]: fidgeting, avoidance, gaze aversion, time spent speaking, and smiling. These behaviors were coded across 6 10 s epochs (i.e., the first 60 s of the speech task), and average scores were calculated for each child’s behavior. The mean values reported represent the average individual 10 s epoch.

#### 2.2.3. Fidgeting

Intensity of nervous fidgeting was coded on a 4-point scale: 0 = *no evidence of fidgeting or very small incidences of fidgeting* to 3 = *highly repetitive and large bodily movements* (e.g., big arm swinging, kicking, and swiveling body back and forth). Intensity of avoidance behavior was coded on a 4-point scale: 0 = *no avoidance behavior* (e.g., the child stands in a designated place, no bodily avoidance) to 3 = *high avoidance behavior* (e.g., the child refuses to stand in a designated place or turns more than 90 degrees, covers their face, and articulates fearfulness).

#### 2.2.4. Gaze Aversion

Gaze aversion was assessed by the extent to which children avoided the gaze of the experimenter and/or the camera over the course of 6 10 s epochs. Each epoch was coded in accordance with either a majority (6 s or more out of 10 s) gaze aversion or social gaze: *gaze aversion* was coded when children averted their gaze from the researcher or camera for at least 6 s within the 10 s epoch; *social gaze* was coded when children sustained their gaze with either the researcher or the camera for at least 6 s within the 10 s epoch. The proportion of epochs in which each child was considered to have been engaging in gaze aversion was then calculated.

#### 2.2.5. Time Spent Speaking

Time spent speaking was assessed by recording the time (in seconds) that the child spent speaking during each 10 s epoch. An average time speaking scare was then calculated across all six epochs.

#### 2.2.6. Smiling

Smiling was coded for each 10 s epoch, and the proportion of epochs in which smiling was present was calculated.

#### 2.2.7. Behavioral Reliability

The Chinese coders who were fluent in both English and Chinese coded behaviors in the Chinese sample. These coders were trained to reach inter-coder reliability with the first author. The coders were naive to the study hypotheses and completed a subset of 20% of participant videos within the Chinese sample to establish inter-rater reliability. The inter-rater reliability was computed on this 20% subset across 2 raters. Inter-rater reliability was established (avoidance: κ = 0.83; fidgeting: κ = 0.82; gaze aversion κ = 0.78; time spent speaking: *r* = 92; smiles: *r* = 0.98). Similarly, two English-speaking RAs coded videos for the Canadian sample on a subset of 19% of the participants (15% subset for time spent speaking). Inter-rater reliability was then established (avoidance: κ = 0.69; fidgeting: κ = 0.73; gaze aversion κ = 0.70; time spent speaking: *r* = 84; smiles: *r* = 0.83).

### 2.3. Data Analysis

A series of Pearson correlations was computed to examine the relations among the five coded shyness-related measures in the country. A repeated measures ANCOVA was conducted to examine the effects of country (China vs. Canada) and gender (boys vs. girls) on five z-scored shyness-related behaviors (gaze aversion, time spent speaking, fidgeting, smiling, and avoidance), while controlling for children’s age. All analyses were performed using SPSS Version 24.

## 3. Results

### 3.1. Descriptive Statistics

Descriptive statistics and between-country differences of the five shyness-related behaviors coded from the birthday speech task are presented in Table 1. The mean values for each behavior measure represent the average per each 10 s coded epoch. Skewness and kurtosis values for these items in both samples were well within the cutoff levels of ±2.

Pearson correlations among the five shyness-related measures for each country are presented in Table 2. Of note, there were significant correlations between gaze aversion and avoidance in both countries, suggesting that these two shyness-related measures were associated with self-presentation in children, regardless of their country. However, the other observed behavioral measures during their speech were not inter-related in either country, suggesting that the individual behavioral measures may have presented different, unrelated, behavioral aspects of shyness.

### 3.2. Country by Gender 

A significant interaction was found between country and gender for shyness-related behaviors, *F* (3.660, 867.521) = 2.850, *p* = 0.027, indicating that the pattern of these shyness-related behaviors differs significantly when considering the interaction of country and gender. In order to understand this interaction, we computed a series of separate two-way ANCOVAs to examine how the interaction between country (China vs. Canada) and gender (boys vs. girls) affected each of the five shyness-related behaviors separately, while controlling for the children’s age (Table 3).

#### 3.2.1. Gaze Aversion

**Figure 1:**
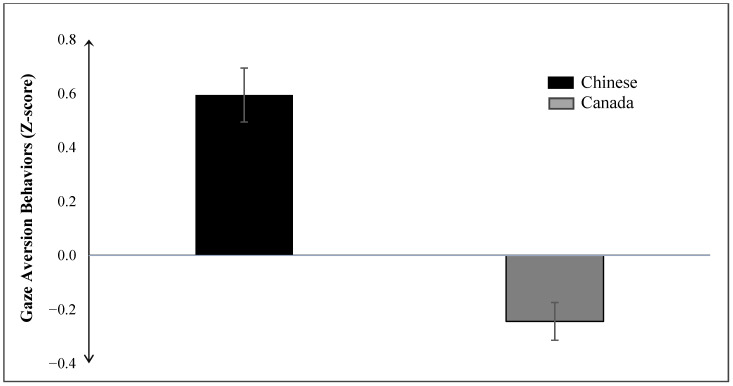
Main effect of country on gaze aversion behaviors. Error bars represent the standard error of the mean.

The results of the ANCOVA reveal that the main effect for country on gaze aversion is significant, *F*(1, 243) = 42.04, *p* < 0.001 (see Figure 1). As predicted, Chinese children (*M* = 0.595, *SE* = 0.10) displayed significantly more gaze aversion compared to Canadian children (*M* = −0.245, *SE* = 0.07). However, the main effect of gender on gaze aversion was not significant, *F*(1, 243) = 2.13, *p* = 0.146, indicating no significant difference in gaze aversion between boys (*M* = 0.031, *SE* = 0.08) and girls (*M* = −0.058, *SE* = 0.09). Moreover, the interaction effect between country and gender on gaze aversion was not significant, *F*(1, 243) = 1.303, *p* = 0.255.

#### 3.2.2. Time Spent Speaking

**Figure 2:**
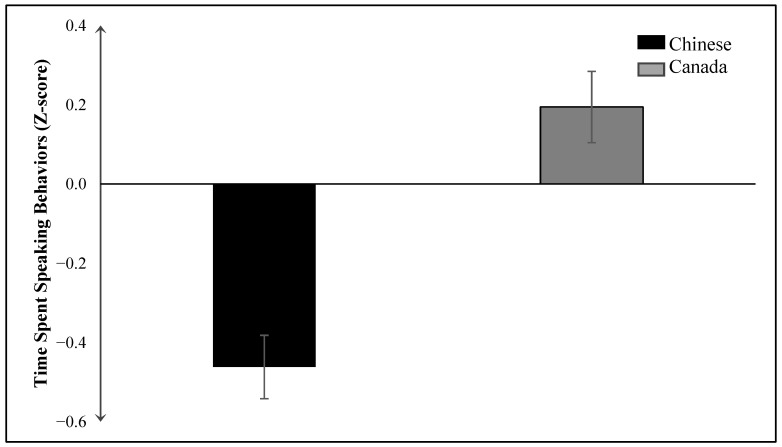
Main effect of country on time spent speaking behaviors. Error bars represent the standard error of the mean.

The results of the ANCOVA reveal that the main effect for country on time spent speaking is significant, *F*(1, 243) = 21.09, *p* < 0.001 (see Figure 2).

As predicted, Chinese children (*M* = −0.46, *SE* = 0.09) spent significantly less time speaking compared to Canadian children (*M* = 0.19, *SE* = 0.07). However, the main effect of gender was not statistically significant, *F*(1, 243) = 0.11, *p* = 0.740, indicating no significant difference in the time spent speaking between boys (*M* = −0.053, *SE* = 0.08) and girls (*M* = 0.089, *SE* = 0.09). Moreover, the interaction effect between country and gender on time spent speaking was not significant, *F*(1, 243) = 0.794, *p* = 0.374.

#### 3.2.3. Fidgeting

**Figure 3:**
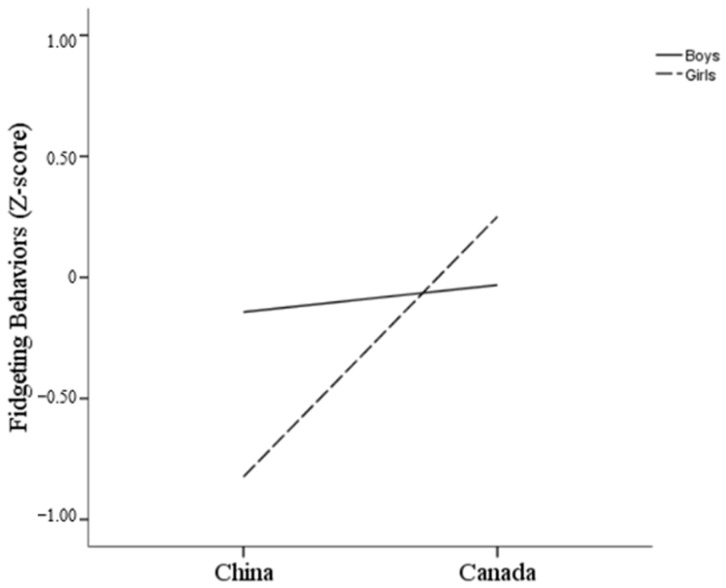
Interaction of country and gender on fidgeting.

The results of the ANCOVA reveal a significant interaction effect between country and gender on fidgeting, *F*(1, 237) = 9.97, *p* = 0.002 (see Figure 3). As shown in Figure 3, this interaction suggests that differences in fidgeting between boys and girls varied by country. Specifically, Canadian girls showed higher fidgeting scores (*M* = 0.25, *SE* = 0.99) compared to Chinese girls (*M* = −0.78, *SE* = 1.10), while Canadian boys had similar fidgeting scores (*M* = −0.038, *SE* = 0.87) compared to Chinese boys (*M* = −0.14, *SE* = 1.02).

#### 3.2.4. Smiling Behaviors

**Figure 4:**
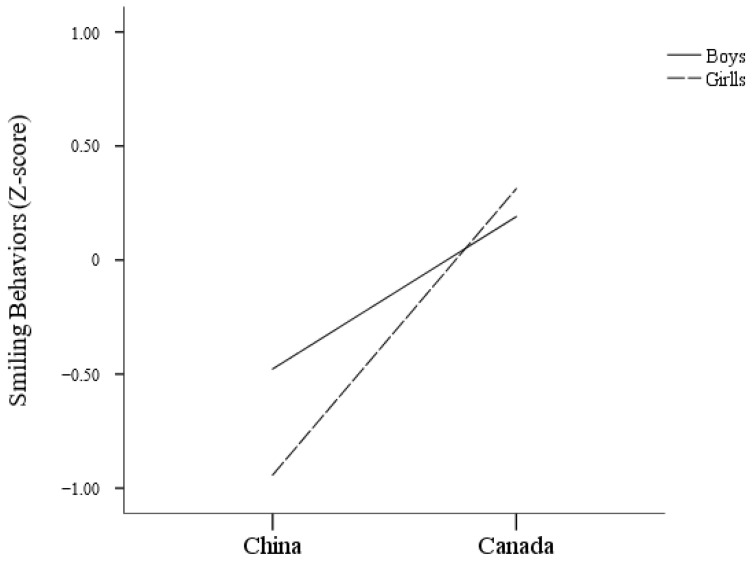
Interaction of country and gender on smiling.

The results of the ANCOVA reveal a significant interaction effect between country and gender on smiling, *F*(1, 242) = 4.00, *p* = 0.047 (see Figure 4). As shown in Figure 4, this interaction suggests that differences in smiling between boys and girls vary by country. Specifically, Canadian girls smiled more (*M* = 0.31, *SE* = 0.09) compared to Chinese girls (*M* = −0.94, *SE* = 0.11), while Canadian boys smiled more (*M* = 0.19, *SE* = 0.10) compared to Chinese boys (*M* = −0.48, *SE* = 0.09).

#### 3.2.5. Avoidance Behaviors

The country and gender interaction effect was not significant, *F*(1,239) = 0.17, *p* = 0.683), nor were the main effects for country, *F*(1, 239) = 0.96, *p* = 0.329 or gender, *F*(1, 239) = 0.31, *p* = 0.577 on avoidance behavior. There was no significant difference in avoidance behavior between Canadian (*M* = −0.046, *SE* = 0.081) and Chinese children (*M* = 0.112, *SE* = 0.097), as well as no significant differences between boys (*M* = −0.042, *SE* = 0.088) and girls (*M* = 0.051, *SE* = 0.093).

## 4. Discussion

In this study, we investigated whether Chinese and Canadian children differed in their observed expressions of shyness-related behaviors in response to a self-presentation task. As predicted, we found that Chinese children exhibited a higher frequency of gaze aversion, lower total time speaking, and a lower frequency of fidgeting and smiling compared with Canadian children during the speech task. Interestingly, the two countries did not differ in the avoidance measure. We also found that many of the observed measures during the speech task were not inter-related in either country, suggesting that the measures might affect different and unrelated behavioral aspects of shyness.

What do between-country and gender differences in children’s observed shyness-related behaviors reflect? Previous studies have shown that Asian groups tend to exhibit more passive, deferential, unconfident, and anxious behaviors in social contexts, whereas Western groups are often characterized as more assertive [14,19]. Western cultures encourage lower levels of shyness, promoting peer acceptance and better well-being, and encouraging children to be more socially assertive [5,36]. In contrast, Eastern cultures emphasize self-restraint and non-assertiveness in interpersonal interactions, prioritizing social harmony [37,38]. Conforming to these social and cultural norms in both cultures is considered crucial for successful social development, even in young children [39]. Our findings support group differences in shyness-related behaviors in Chinese and Canadian children during a self-presentation task and suggest that culture may play a role in shaping these differences [18].

Eye contact can hold different cultural meanings regarding its directness [40]. In Western cultures, direct eye contact during communication is often encouraged, whereas in Eastern cultures, influenced by the etiquette values of Confucianism, direct eye contact is considered impolite and should be avoided when communicating [41]. Thus, this may be one explanation for the higher level of gaze aversion in the Chinese compared to the Canadian children. Researchers have posited that people who unknowingly attribute certain motives to eye avoidance (e.g., inattention, rudeness, shyness, and low intelligence) tend to be more biased [42].

Regarding the length of speech, previous research has supported the idea that children who are consistently shy and reticent are considered well-behaved in China [43]. Similarly, sensitive and silent children are considered “*guai*”, a common term used to praise children in China [43]. Therefore, Chinese children may be more likely to exhibit silent behavior due to Chinese acceptance than Canadian children.

The overall trend shows that fidgeting and smiling are relatively similar across cultures, with children from the same country exhibiting similar patterns. However, the differences in these behaviors between girls from China and Canada are more obvious than those observed between boys. This significant interaction between country and gender suggests that cultural expectations and norms play a critical role in shaping how boys and girls express discomfort or anxiety in social situations. In Canada, girls exhibited higher levels of fidgeting and smiling compared to their Chinese counterparts, while boys from both countries showed similar levels of fidgeting. Although Canadian boys smiled more frequently than Chinese boys, the difference was less obvious compared to that observed in girls. These patterns can be understood through the lens of cultural differences in the socialization of emotional expression.

In Chinese society, Confucianism plays an important role in influencing fidgeting behaviors, emphasizing the importance of considering others’ views and evaluations [44,45]. As a result, Chinese children may be more susceptible to social threats in evaluative contexts, leading to fear-related behaviors, such as freezing or avoidance [46]. Therefore, Chinese children fidgeted less than Canadian children. Additionally, Chinese culture places greater social expectations on girls to behave delicately and quietly [28], which may explain why Chinese girls exhibit lower levels of fidgeting compared to boys.

Cultural differences in smiling behaviors can also be attributed to distinct norms and attributions regarding emotional expression. Researchers indicated that Canadian smile attributions may be based on internal emotional responses, while Chinese smile attributions may be based on modesty and assurance of the other person’s face [47]. Therefore, these distinct cultural norms provide insights into why Chinese children may not smile as frequently in social settings. In Western cultures, smiling is often encouraged as a social behavior, especially for girls, who are typically socialized to be warm, friendly, and approachable [48]. This could explain why Canadian girls smile more compared to Chinese girls, who may be subject to cultural norms that emphasize modesty and emotional restraint [49,50,51]. For boys, while there is also the encouragement to smile in the Canadian culture, this expectation may not be as strongly enforced as it is for girls, leading to a smaller difference in smiling behavior between Canadian and Chinese boys. Therefore, although Chinese children generally exhibit lower levels of fidgeting and smiling compared to Canadian children, these differences are more pronounced among girls. This suggests that cultural norms place greater emphasis on behavioral expectations for girls, leading to more significant variations in how these behaviors are expressed across cultures.

Lastly, it is noteworthy that, although the mean level of avoidance behavior was higher in Chinese children than Canadian children, the two groups were not significantly different from one another regarding this behavioral measure. This may be due to the smaller sample size of Chinese children limiting the frequency of the behavior for this particular measure. Another possible explanation is that slight discrepancies in the positions of the experimenters and cameras between China and Canada resulted in deviations in the coding of avoidance behavior due to differences in the control of angles.

### Strengths, Limitations, and Future Directions

The present study had several noteworthy strengths. These included (1) the collection of shyness-related behaviors from direct observations rather than a reliance on self-, teacher, and/or parent reports; (2) the developmental age studied, which coincides with the maturation and emergence of reliably measured social shyness; and (3) a direct comparison of children who were raised and residing in different countries, rather than comparing cultural differences among different cultural groups residing in the same country.

Despite these strengths, our study also had several limitations. First, unlike previous recent studies with self-report measures [52], we were unable to test for measurement invariance between the two countries to see if the coded measures were methodologically equivalent between China and Canada because of the relatively small sample sizes. Second, despite the advantages of observational methods, the micro-level behavioral variables chosen for coding in this study may not be equally sensitive to shyness, social anxiety, or social evaluation across ethnic groups [13]. Third, even though reliability among behavioral coders was established within and between countries, there may have been unmeasured variables and factors due to cultural differences in a setting that we did not control that may have adversely affected our observations. For example, the size of the observation room and the positioning of experimenters and cameras need to be considered. Fourth, the homogeneity of variance assumption was not met for smiling and avoidance behaviors, as indicated by Levene’s test. This was likely due to cultural norms and individual coping strategies influencing these behaviors, leading to greater variability. Therefore, we need to exercise caution when interpreting the results for these two measures. Future research could use more robust statistical analyses to better understand cultural variances and may consider using alternative coding approaches and implementing more precise study designs to ensure consistency in behavioral observations. Future research should involve larger and more demographically diverse samples that incorporate multiple measurements of shyness in different contexts and establish measurement invariance for the measures to ensure trustworthiness between country comparisons. Future work should also consider examining psychophysiological measures between countries to extend and provide convergent evidence to the present findings. In terms of practical implications, it is important to consider how these findings can inform educational and social interventions, particularly for children transitioning between cultures. For example, when children from different cultures migrate or travel to new cultural contexts, understanding how cultural differences might condition the expression of shyness can be crucial for both educators and parents. Providing supportive environments that respect cultural differences in emotional expression may help children adapt more comfortably to new cultural settings and improve their social understanding.

## 5. Conclusions

To our knowledge, no previous studies have directly compared observed shyness-related behaviors in children in response to a self-presentation speech task at this age from Eastern and Western cultures. Our preliminary findings extend prior work examining cross-cultural differences in children’s shyness using subjective measures of self-, teacher, and parent reports to measures obtained from direct observation. This study demonstrates the impact of culture on the expression of some shyness-related behaviors in children, highlighting the importance of cultural norms and individual behaviors. These findings also contribute to our understanding of how cultural context influences the manifestation of shyness-related behaviors in social contexts and also provide insights for educators, parents, and researchers interested in promoting the socioemotional development of children from diverse cultural backgrounds.

## Figures and Tables

**Table 1:** Means and standard deviations and between-group differences on the five expressions of children’s shyness-related behaviors by country.

	China (*N* = 74)				Canada (*N* = 189)						
Variable	*M*	*SD*	Skewness	Kurtosis	*M*	*SD*	Skewness	Kurtosis	*Df*	*t*	*p*
Gaze aversion	0.740	0.278	−0.754	−0.529	0.472	0.306	0.049	−0.984	250	6.504	<0.001
Time spent speaking	3.449	2.232	0.659	−0.251	4.985	2.206	−0.290	−0.543	250	−5.017	<0.001
Fidgeting	1.639	0.770	0.101	−0.935	1.900	0.664	−0.554	0.084	119.420	−2.528	0.013
Smiling	0.232	0.326	1.285	0.370	0.578	0.388	−0.315	−1.479	161.566	−7.216	<0.001
Avoidance	1.667	0.586	0.257	−0.591	1.557	0.753	−0.226	−0.867	174.885	1.234	>0.05

Notes. Mean values represent the mean value in an average 10 s coded epoch. Between country differences are evaluated at *p* = 0.01 to correct for multiple comparisons (i.e., *p* < 0.05/5 = *p* = 0.01).

**Table 2:** Correlations among the five expressions of children’s shyness-related behaviors by country.

Item	1	2	3	4	5
1. Gaze aversion	-	−0.026	−0.208	−0.102	0.498 **
2. Time spent speaking	0.106	-	−0.018	0.021	−0.034
3. Fidgeting	−0.045	0.013	-	0.199	0.203
4. Smiling	−0.157 *	−0.002	0.256 **	-	0.140
5. Avoidance	0.262 **	−0.021	0.272 **	−0.021	-

Notes. Correlations above the diagonal are for the Chinese sample, and correlations below the diagonal are for the Canadian sample. All behaviors are standardized. Chinese *N* = 74; Canadian *N* = 189. * *p* < 0.05; ** *p* < 0.01.

**Table 3:** Results of repeated measures ANCOVA for the effects of country, gender, and age on shyness-related behaviors.

Source	Sum of Squares	*df*	Mean Square	*F*	*p*	Partial *η*^2^
**Within subjects**						
Behaviors	24.357	3.66	6.654	7.325	<0.001	0.030
Behavior × Country	93.172	3.66	25.454	28.020	<0.001	0.106
Behavior × Gender	4.634	3.66	1.266	1.394	>0.05	0.006
Behavior × Country × Gender	9.476	3.66	2.589	2.850	0.027	0.012
Error (behaviors)	788.068	867.521	0.908			
**Between subjects**						
Country	9.834	1	9.834	8.731	0.003	0.036
Gender	0.069	1	0.069	0.061	0.805	0.000
Country × Gender	5.027	1	5.027	4.463	0.036	0.018
Age (Covariate)	0.461	1	0.461	0.409	0.523	0.002
Error	266.954	237	1.126			

## Data Availability

The data presented in this study are available on request from the corresponding authors.
