# Correction: Kong et al. (2024). Observed Shyness-Related Behavioral Responses to a Self-Presentation Speech Task: A Study Comparing Chinese and Canadian Children. *Behavioral Sciences*, *14*(12), 1147

**DOI:** 10.3390/bs15050680

**Published:** 2025-05-16

**Authors:** Xiaoxue Kong, Taigan L. MacGowan, Shumin Wang, Yan Li, Louis A. Schmidt

**Affiliations:** 1 Department of Psychology, University of Northern British Columbia, Prince George, BC V2N 4Z9, Canada; 2 Department of Psychology, Neuroscience & Behaviour, McMaster University, Hamilton, ON L8S 4L8, Canada; schmidtl@mcmaster.ca; 3 Department of Psychology, Queen’s University, Kingston, ON K7L 3N6, Canada; macgowat@mcmaster.ca; 4 Shanghai Institute of Early Childhood Education, Shanghai Normal University, Shanghai 201418, China; 1000529027@smail.shnu.edu.cn (S.W.); liyan@shnu.edu.cn (Y.L.)

In the original publication (Kong et al., 2024), there were two mistakes in Figure 3 and the citation of Table 1 as published. 

1. The Figure 3 duplicates Figure 4 and needs to replace Figure 3 with a new version. The corrected Figure 3 appears below. 

**Figure 3:**
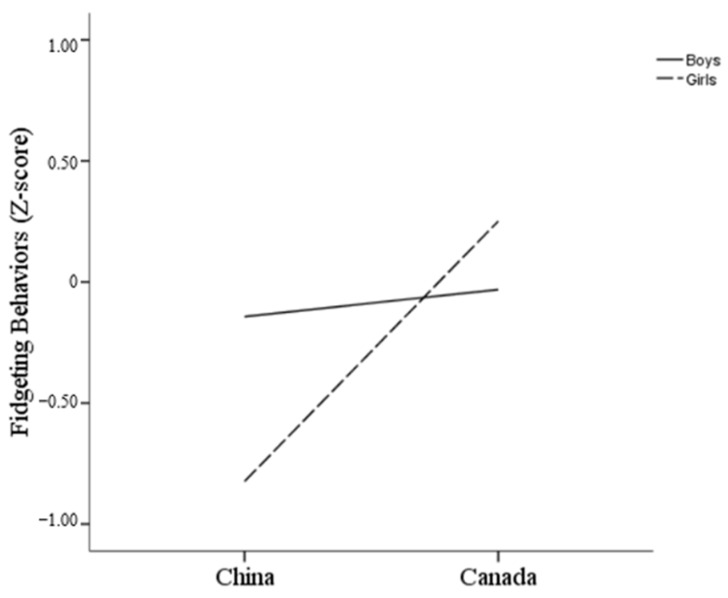
Interaction of country and gender on fidgeting.

2. There is a sentence, which is “All behaviors are standardized”, in the Table 1’s notes that needs to be deleted. The corrected Table 1 appears below.

**Table 1:** Means and standard deviations and between-group differences on the five expressions of children’s shyness-related behaviors by country.

	China (*N* = 74)				Canada (*N* = 189)						
Variable	*M*	*SD*	Skewness	Kurtosis	*M*	*SD*	Skewness	Kurtosis	*Df*	*t*	*p*
Gaze aversion	0.740	0.278	−0.754	−0.529	0.472	0.306	0.049	−0.984	250	6.504	<0.001
Time spent speaking	3.449	2.232	0.659	−0.251	4.985	2.206	−0.290	−0.543	250	−5.017	<0.001
Fidgeting	1.639	0.770	0.101	−0.935	1.900	0.664	−0.554	0.084	119.420	−2.528	0.013
Smiling	0.232	0.326	1.285	0.370	0.578	0.388	−0.315	−1.479	161.566	−7.216	<0.001
Avoidance	1.667	0.586	0.257	−0.591	1.557	0.753	−0.226	−0.867	174.885	1.234	>0.05

Notes. Mean values represent the mean value in an average 10 s coded epoch. Between country differences are evaluated at *p* = 0.01 to correct for multiple comparisons (i.e., *p* < 0.05/5 = *p* = 0.01).

The authors state that the scientific conclusions are unaffected. This correction was approved by the Academic Editor. The original publication has also been updated.

## References

[B1-behavsci-15-00680] Kong X., MacGowan T. L., Wang S., Li Y., Schmidt L. A. (2024). Observed Shyness-Related Behavioral Responses to a Self-Presentation Speech Task: A Study Comparing Chinese and Canadian Children. Behavioral Sciences.

